# Government health expenditure in India

**DOI:** 10.2471/BLT.14.143362

**Published:** 2015-04-27

**Authors:** Manoj Grover

**Affiliations:** a Health and Family Welfare Division, Planning Commission (NITI Aayog), Government of India, Yojana Bhavan, Sansad Marg, New Delhi, 110001, India.

In their paper about progress towards universal health coverage in Brazil, the Russian Federation, India, China, South Africa (BRICS),^1^ Rao et al. report that in India in 2011, of total government expenditure, about 8% was expended on health. This estimate was from the World Health Organization’s (WHO’s) global health observatory data respository,^2^ which is based on national health accounts. For India, data on the national health accounts were last collected in 2004–2005. More recent data are usually based on estimates obtained through national technical contacts or from publicly-available documents, which are then harmonized with the national health accounts framework. Missing values are estimated using various accounting techniques depending on the national data available.^3^

However, according to the Indian Government’s budget and expenditure data for the fiscal year 2012–2013 (Table 1),^4^^–^^7^ the government spent about 1 104 543 million rupees on health (central and states combined). This is about 3.68% of the total government expenditure of 30 037 588 million rupees and not 8% as quoted in the article.^1^

**Table 1:** Government health expenditure in India, 2012–2013

Expenditure	State (revised estimate)			Central (actual expenditure)		Total
	Plan^a^	Non-plan^b^		Plan^a^	Non-plan^b^	
**Overall, million rupees**	5 911 346	11 111 429		3 047391	9 967 422	**30 037 588**
**Health, million rupees**	335 562	436 952		268 667	63 362	**1 104 543**
**% spent on health**	5.68	3.93		8.82	0.64	**3.68**

^a^ Plan expenditures include new projects and/or completion of projects started in the last five years.

^b^ Non-plan expenditures are obligatory expenditures such as maintenance, interest payments, subsidies and pensions.

Notes: Revised estimates also include supplementary requests for funds (during the financial year) from the state government, in addition to the budget estimate. Revised estimates are listed separately in the data sources. Central assistance for state and union territory plans have been included under the state plan expenditures and excluded from the central government’s expenditure.^4^^,^^5^

Data sources: State government^6^ and central government.^4^^,^^5^^,^^7^
